# Design of the Anti-tuberculosis Drugs induced Adverse Reactions in China National Tuberculosis Prevention and Control Scheme Study (ADACS)

**DOI:** 10.1186/1471-2458-10-267

**Published:** 2010-05-21

**Authors:** Yin Yin Xia, Dai Yu Hu, Fei Ying Liu, Xiao Meng Wang, Yan Li Yuan, De Hua Tu, Yi Xin Chen, Lin Zhou, Li Zhen Zhu, Wei Wei Gao, Hong Yuan Wang, Da Fang Chen, Li Yang, Ping Ping He, Xiao Ting Li, Ying Jian He, Feng Sun, Si Yan Zhan

**Affiliations:** 1 Department of Epidemiology and Biostatistics, School of Public Health, Peking University, Beijing, China; 2 Center for Disease Control and Prevention in Chongqing Municipality, Chongqing, China; 3 Center for Disease Control and Prevention in Guangxi Zhuang Autonomous Region, Nanning, China; 4 Center for Disease Control and Prevention in Zhejiang Province, Hangzhou, China; 5 Center for Disease Control and Prevention in Jilin Province, Changchun, China; 6 Beijing Institute for Tuberculosis Control, Beijing, China; 7 Center for Drug Reassessment, State Food and Drug Administration, Beijing, China; 8 Center for Tuberculosis Control and Prevention, Chinese Center for Disease Control and Prevention, Beijing, China; 9 Beijing Tuberculosis and Thoracic Tumor Research Institute, Beijing, China; 10 Department of Health Policy and Management, School of Public Health, Peking University, Beijing, China

## Abstract

**Background:**

More than 1 million tuberculosis (TB) patients are receiving the standard anti-TB treatment provided by China National Tuberculosis Prevention and Control Scheme (CNTS) in China every year. Adverse reactions (ADRs) induced by anti-TB drugs could both do harm to patients and lead to anti-TB treatment failure. The ADACS aimed to explore ADRs' incidences, prognoses, economical and public health impacts for TB patients and TB control, and build a DNA bank of TB patients.

**Methods/Design:**

Multiple study designs were adopted. Firstly, a prospective cohort with 4488 sputum smears positive pulmonary tuberculosis patients was established. Patients were followed up for 6-9 months in 52 counties of four regions. Those suspected ADRs should be checked and confirmed by Chinese State Food and Drug Administration (SFDA). Secondly, if the suspected ADR was anti-TB drug induced liver injury (ATLI), a nested case-control study would be performed which comprised choosing a matched control and doing a plus questionnaire inquiry. Thirdly, health economical data of ADRs would be collected to analyze financial burdens brought by ADRs and cost-effectiveness of ADRs' treatments. Fourthly, a drop of intravenous blood for each patient was taken and saved in FTA card for DNA banking and genotyping. Finally, the demographic, clinical, environmental, administrative and genetic data would be merged for the comprehensive analysis.

**Discussion:**

ADACS will give an overview of anti-TB drugs induced ADRs' incidences, risk factors, treatments, prognoses, and clinical, economical and public health impacts for TB patients applying CNTS regimen in China, and provide suggestions for individualized health care and TB control policy.

## Background

Tuberculosis (TB) is one of the most common infectious diseases globally. According to WHO reports, there were an estimated 9.3 million incident cases and 13.7 million prevalent cases of TB in 2007[1]. China is the second highest TB burden country in the world, only next to India. To control TB epidemic, China established China National Tuberculosis Prevention and Control Scheme (CNTS) in 1990, and started implementing directly observed treatment strategy (DOTS) since 1991. Nowadays DOTS has covered all population in China [1], and became the major TB control access. The key component of DOTS strategy is the standard anti-TB short course chemotherapy regimen. The regimen which requires continually taking drug combinations of Isoniazid(H), Rifampicin (R), Pyrazinamide(Z), Ethambutol(E) and Streptomycin(S) every other day for 6-9 months, is recommended by WHO and currently used in the majority of high TB burden countries[2]. With loans and grants from World Bank and UK Department for international development, Chinese government promises that all sputum smear positive TB patients could get the standard therapy freely from local Center for Disease Control and Prevention (CDC) [3].

Patient should come to CDC to get drugs monthly and take drugs at home with direct observation by a village or town clinic doctor as the supervisor. Nowadays, nearly 100,000 new detected smear positive TB patients are going through this therapy every year [1]. Drugs in the therapy (H, R, Z, E, S), in addition to their role of killing and containing Mycobacterium effectively, could cause different kinds of adverse reactions (ADRs), such as hepatotoxic reaction, gastro-intestinal discomfort, drug allergy, arthralgia, etc. Those ADRs are regarded as one of the major causes of incompliance of anti-TB treatment [4,5]. They may lead to final termination of TB treatment and severe ADRs outcomes like liver failure or death as well. As to the ADRs overall incidence, no consensus has been reached. Different studies may vary from 5.5% to 57.8% according to different populations and ADRs definitions [6-11]. Generally speaking, studies with larger sample tend to report relatively lower incidence which may be related to their retrospective study design. In China, our previous systematic review showed that the integrated ADRs overall incidence was around 12.6% [12]. But most of individual studies in our systematic review had small sample size, oversensitive ADRs diagnostic standards and were done in general hospitals. Very few studies focused on CNTS therapy induced ADRs.

The possible environmental and genetic factors of anti-TB induced ADRs have always been the matter of concern. It is well documented that the risk of ADR increases with age [13-16], malnutrition [17-20], and history of hepatitis [16,21]. In addition, a large number of other environmental factors have been hypothesized to be associated with ADRs such as human immunodeficiency virus infection [20,22-25] and hepatitis C virus infection [22]. But questions remain about the effects of these exposures either because previous studies have shown mixed results (Caucasian[26], gender[26,27], high alcohol intake[18,28], Hepatitis B virus infection[15,29-31]) or because they have been retrospective and susceptible to a variety of biases (Severity of TB[29,32]). Genetic factors like isoniazid-metablising enzyme gene polymorphisms were studied a lot but these studies also showed inconsistent results [33]. Until now, the comprehensive study for environmental, genetic, clinical and administrative factors has not been reported.

Besides, there is a typical clinical practice in China: prescribing liver protected drugs to patients before they initiate the anti-TB treatment in order to lower the risk of hepatotoxicity. Usually those drugs will be taken for 2 months or through the whole anti-TB treatment. A number of drugs using for curing or relieving symptoms of drug-induced hepatitis are used in this kind of prevention although neither their effects nor safety have been convincingly approved in preventive use[34]. It brings patients extra economical burden, yet with unconfirmed effect and risks of new ADRs induced by those liver protective drugs. We intended to study those environmental, genetic and clinical factors comprehensively to help develop individualized anti-TB regimen in the future and assess the necessity of preventive drug use.

Although it is well known that those ADRs will do great harm to patients, the economical impacts of ADRs are seldom studied. Different kinds of ADRs call for different kinds of examinations and treatments which differ in costs. Hospitalizing, transport, nutrition supplement, loss of working time and anti-TB drug replacements will also lead to considerable amount of costs. Another negative impact of anti-TB drug induced ADR is that patient may have regimen change, regimen suspension or temporarily interruption after ADR happens. Thus it may not only cause personal anti-TB treatment failure, but will also affect TB epidemic control since the patient still remains in Mycobacterium transmittable status and could possibly infects 10-15 more people in 12 months[35]. Therefore, getting an overview of those clinical, economical and public health impacts for TB patients and TB control will be quite meaningful.

## Methods/Design

### Overview and objectives

The ADACS is a prospective longitudinal study of anti-tuberculosis drugs induced adverse reactions based on a multi-center cohort of 4488 pulmonary tuberculosis patients receiving China National Tuberculosis Prevention and Control Scheme treatment.

The aim of ADACS was to recognize incidences, prognoses, impacts, and risk factors of anti-tuberculosis drugs induced ADRs in CNTS, and provide individualized health care and TB control policy suggestions. The major objectives of ADACS were:

1) To determine the incidences of anti-tuberculosis drugs induced ADRs in CNTS and the harm to TB patients in China.

2) To evaluate the impacts of anti-tuberculosis drugs induced ADRs on China tuberculosis control.

3) To explore genetic and environmental risk factors relevant with anti-tuberculosis induced ADRs and their interactions.

4) To investigate the usage and effect of liver protective drugs.

5) To evaluate the health economic burden of anti-tuberculosis induced ADRs and their treatments.

6) To build a TB patient DNA samples bank for future research.

### Study area selection

The study fields were selected to meet those criteria: 1) including provinces located in north and south, west and east of China; 2) including provinces comparatively developed and developing in economic status; 3) the selected provinces had performed DOTS well; 4) CDC of counties in the selected provinces had laboratories to do the study required examinations independently.

**Figure 1:**
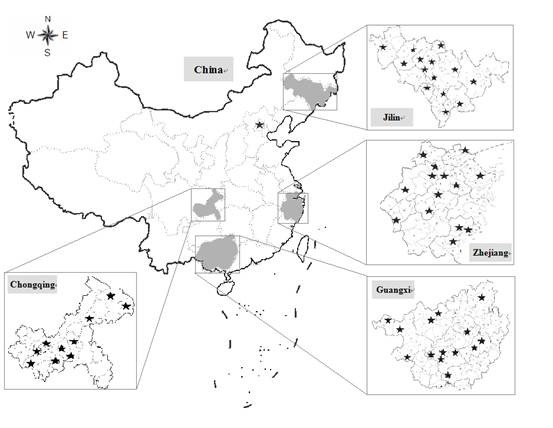
**Geographic map of study fields in ADACS**.

Four districts, Zhejiang Province, Jilin Province, Guangxi Zhuang Autonomous Region, and Chongqing Municipality were chosen ultimately with expert consultancy. Zhejiang, Guangxi, Chongqing represented south of China, Jilin represents north of China; Zhejiang represented east of China, Guangxi and Chongqing represented west of China; Zhejiang represented economic developed districts and Chongqing, Jilin, Guangxi represented economic developing districts according to their gross domestic product(GDP) contribution in 2006[36]. All of the 4 districts had performed DOTS strategy well, had educated personnel and equipped laboratories. Figure 1 shows precise locations of the 4 districts and sampled counties. The total number of sampled counties was 52, 14 for each in Guangxi, Jilin, Zhejiang, and 10 in Chongqing. The counties were chosen randomly from all eligible counties in each setting district.

### Sample size assessment

We adopted stratified, cluster and probability proportional to size (PPS) sampling strategy. For the start, we calculated a sample size under the assumption of simple random sampling in infinite population, using parameters and formula as follows:

P: The expected incidence of overall ADRs, 12% was used according to our previous systematic review [12].

*d: *Absolute Precision, here we took 2%.

1-*α*: Confidence level, we set 1-*α *= 95%, and *u*_*α *_= 1.96.

Then to correct for the cluster sampling design, n_1 _was multiplied by the design effect (*deff*). We assumed the average size of the clusters M was 80. But since we couldn't find reliable assumption of interclass correlation coefficient in TB patients group, we couldn't calculate *deff *precisely, so it was simply set to be 2.

Once again we enlarged n_2 _by 2 for we might need to do stratified analysis of some binary variables after all research data would have been collected.

Finally, the number was increased by 10% considering expected losses.

So the ultimate anticipatory sample size was determined to be 4500 and the number of cluster n_c _was approximately 56. We grouped 4 districts into 4 strata, and then allocated 14 clusters (counties) and 1125 patients to each stratum. But since Chongqing Municipality has fewer counties for randomising, its clusters were reduced to 10 and the total clusters were 52. In the end, the average size of our clusters was 87. In each county how many participants should be sampled was then decided using PPS without replacement sampling technique, in accordance with the proportion of number of the counties' new reported sputum smear-positive TB patient in 2006.

### Participant eligibility and recruitment

The study recruitment began in October 2007 and finished by June 2008. In the sampled 52 counties, the local CDC investigators recognized all primary or re-treatment sputum smear positive pulmonary TB patients who would accept CNTS free treatment there as potentially eligible participants. If patients met the inclusion and exclusion criteria (Table 1), investigators would explain the study to those patients, invite them for participation. If the patients were eligible but not recruited, reasons should be recorded. During the follow up, if patients took a withdrawal from the study actively or passively in accordance with the withdrawal criteria, reasons would also be logged.

**Table 1:** Inclusion, exclusion and withdrawal criteria for participants in the ADACS program

Criteria	Detailed items
Inclusion criteria	1. Primary or re-treatment sputum smear positive pulmonary tuberculosis patients; 2. Receiving standardized short course chemotherapy recommended by CNTS in local CDC; 3. Willing to join the study and signing the informed consent by himself or surrogate.
Exclusion criteria	1. Having psychiatric disease which induces incorporation of questionnaire investigation; 2. Having severe diseases with prognosis shorter than 6 months; 3. Having certain problems with signing informed consent;
Withdrawal criteria	1. Unwilling to keep on participating in the study; 2. Incompliance like stopping taking CNTS drugs more than 2 months; 3. Developing diseases in the exclusion criteria after enrollment; 4. Out-migrating or temporarily going out, missing the scheduled laboratory examination; 5. Death which is not caused by anti-TB drugs induced ADR.

During the recruitment phase, 6460 smear positive patients were recognized in our 52 study fields, 6305 patients were eligible and 4488 of them were recruited. The participation rate of eligible subjects was 71.2%. Number of primary and re-treatment TB patients were 3820 and 668 respectively.

### Follow-up procedures

**Figure 2:**
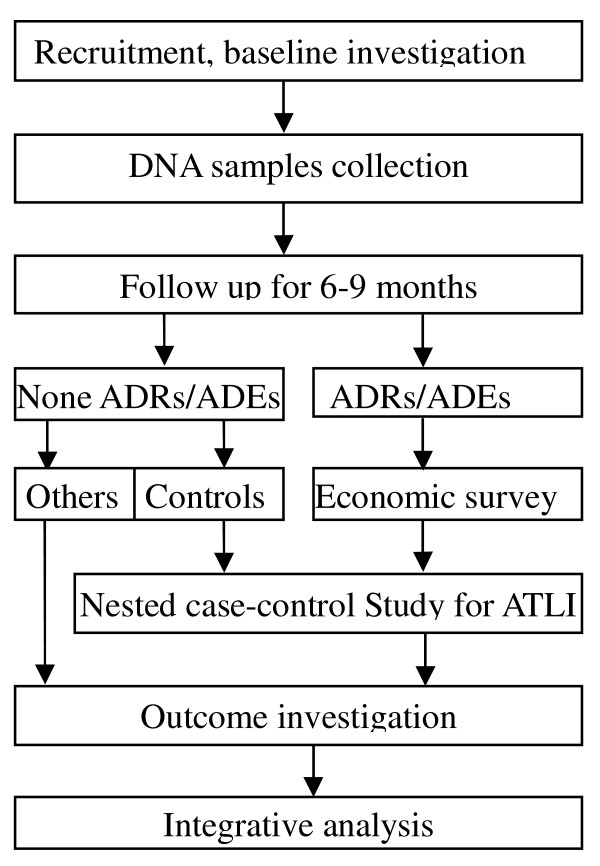
**Flow chart of ADACS implementing scheme**.

The whole study scheme is demonstrated by figure 2. Several phases of data collection were envisaged for this study.

Firstly, participants recruited would finish the baseline questionnaire and receive several physical and laboratory examinations, including weight, height measurement, blood, urine routine test, liver, renal function test and hepatitis B surface antigen (HBsAg) test. All tests were done in local CDC laboratory using uniform reagents and equipments corrected with standard sample from National CDC lab. A drop of intravenous blood for each patient from the laboratory examination blood sampling was taken and saved in Whatman FTA card [37] for future genetic analysis and DNA banking.

Then a follow up for 6-9 months was started. For primary TB patients it was 6 months, while for re-treatment TB patients it was 8 months. The first 2 months were defined as initial intensive phase, in which primary and re-treatment patients would take HRZE and HRZES combination respectively. The afterwards 4 or 6 months were consolidation phase, in which they would take HR and HRE combination respectively. If patient extended intensive phase by 1 more month, follow up period would prolong 1 month correspondingly. Since basically every patient would come to local CDC monthly to pick up their anti-TB drugs for next month' use, the local CDC doctors could get direct contact with them at least once a month and receive reports from supervising doctors from time to time.

One month after anti-TB treatment started, patients should take blood routine test, liver and renal function test again. For all participants, ADACS program provided these 2 times of free laboratory examinations. Participants with suspicious ADRs who were referred as adverse drug events (ADEs) patients would have another free examinations after ADEs happened. In spite of those scheduled examinations, patients might also take medical tests of other kinds or in other times personally in general hospitals, their results would be recorded and assessed in a later stage to be decided approved or not.

During the follow up period, patients should fill in their ADACS calendars which were designed to record patients' everyday feelings and their drug usages. Once patients had unbearable discomfort, they should report it to their supervising doctors or CDC doctors, do examinations needed in time. According to DOTS, TB patients' supervising doctors were supposed to watch patients taken their drugs every other day although in some places they might only do this once a week. They would supervise patients' drug reactions and check their ADACS follow up calendars. If they assumed patients may have ADRs, they would refer patients to CDC doctors for further examinations and ADEs investigations. On the other hand, if a patient didn't develop any discomfort, but his scheduled examinations showed abnormity, the ADEs investigation procedure would also be activated.

For identifying anti-TB induced ADEs, we had cooperation with Center for Drug Reassessment (CDR) of Chinese SFDA and adopted their principle, "report on suspicious". That meant an ADE would be reported under two conditions. The first one was when a patient developed sign or symptom which called for medication. The second one was the patient did not need medication but altered his anti-TB treatment due to ADE. The ADE would be reported to both study investigator and National ADR monitoring system online. SFDA CDR would then check the report, evaluate the causality of drugs and events, approve it to be an ADR or just an ADE. The causality assessment was following standards of WHO Uppsala Monitoring Centre system [38], which mainly based on drug characteristic, time sequence of drug taken and adverse event, reaction of cutting dose, stopping or reintroducing the suspicious drug and existence of other possible causes. Ultimately the causality of ADE and anti-TB drug would be labeled as certain, probable/likely, possible, unlikely, conditional/unclassified or unassessible/unclassifiable. For ATLI, there was a detailed grading diagnosis criteria which were produced by SFDA CDR, based on Alanine transaminase (ALT) performance.

After an ADE was identified, investigator should trace and inquire patient about ADE treatment, prognosis, impaction on anti-TB treatment drug change, drug withdrawal, then fill them in the study file. Economical data should be collected around 1 month after ADE happened to make sure that medication costs had been paid, yet still remembered clearly. Medical records and charging bills of both outpatient visit and hospital stay should be checked. Health insurance payment and self payment should be distinguished and recorded. Those treatment, prognosis, impact and cost inquiries should be done again when the whole anti-TB therapy ended. Different ADEs in one person would be recorded separately. Same ADE in different time in one person which was proved to be re-occurrence would also be recorded separately.

If the suspected ADR was ATLI, a nested case-control study would be performed which comprised choosing a matched control and doing a plus questionnaire inquiry for both ATLI patient and control. The selection of control complied with 3 rules: 1) same sex with case; 2) age difference from case less than 5 years; 3) primary or re-treatment patient as case. The plus questionnaire has 3 components: surveillance of TB treatment, patient's lifestyle risk factors, and patient's knowledge of TB and their faith in current treatment.

When patient finished the standard anti-tuberculosis therapy, their tuberculosis outcomes were recorded. The blood samples collected previously were processed in the central laboratory in Peking University for genotyping.

### Data collection

During the study, most information was obtained by interviewing participants in person by the local CDC investigators. The whole process of TB treatment and its outcome were also recorded by CDC investigators. Physical, laboratory examinations and genotyping results were logged by lab technicians from CDC and Peking University. Our main investigation instruments and variables are summarized in table 2 and table 3.

**Table 2:** Main investigation instruments and their components of ADACS-1

Instruments	Categories	Variables
Baseline questionnaire	Demographic characteristics	Age; Sex; Nationality; Education; Occupation; Marriage; Family income of last year; Health insurance, etc.
	Family history	Liver, renal and other disease history of family members.
	Medical history and concurrent disease	Previous hepatitis history; Previous hepatitis virus tests results. Diagnosis and duration of past renal, visional, auditive and other chronic disease history; Allergic history; Concurrent chronic diseases.
	TB diagnosis and previous treatment	Definitive diagnosis of TB; Diagnosis time; Diagnosis agency; Anti-TB treatment history; Anti-tuberculosis induced ADEs occurrence history.
	Current drug usage information	Product name; Production NO; Dose; Method and cost of anti-tuberculosis drugs and other concurrent using drugs.
Physical, laboratory examination	Physical examinations	Height; Weight.
	Blood routine test	Erythrocyte count (10^12^/L); Leukocyte count (10^9^/L); Hemoglobin concentration (g/L); Platelet count (10^9^/L).
	Urine routine test	PH value; Urine protein; Erythrocyte count; Leukocyte count; Urinary cylinder count.
	Liver and renal function test	Alanine aminotransferase (ALT, U/L); Aspartate aminotransferase (AST, U/L); Total bilirubin (TBIL, μmol/L); Blood urea nitrogen (BUN, mmol/L).
	Hepatitis B virus marker	HBsAg test.
Medication record	Anti-TB drugs	Name, commercial name, manufacturer, production number, usage, dosage, starting and ending time of anti-TB drugs, including RHZES and other supplementary anti-TB drugs.
	Liver protective drugs	Name, commercial name, manufacturer, production number, usage, dosage, starting and ending time of drugs used for preventing ATLI.
	Other drugs	Name, commercial name, manufacturer, production number, usage, dosage, starting and ending time of other drugs used during the anti-TB therapy, for example, antihypertensive drugs, hypoglycaemic drugs, etc.

**Table 3:** Main investigation instruments and their components of ADACS-2

Instruments	Categories	Variables
ADE assessment and investigation questionnaire	ADE occurring features	ADE affected organs; Signs and symptoms; Symptoms first appearing time and their duration; Suspected drugs and their sequencing; Assessment of ADE relationship with anti-TB drugs.
	ADE treatment, prognosis	Clinical consultation, treatment, examination and hospitalization induced by ADE; Drugs used; Examinations taken; Days of hospitalization; ADE prognosis.
	Direct and indirect costs of ADE	Drug, examination, hospitalization cost; Transport, nutrition, nursing fees; missing working time of patients and family; Impaction on anti-TB treatment such as drug change, drug withdrawal and costs induced; Percentage of health insurance payment for direct medication expenditure.
Nested case-control questionnaire	TB treatment surveillance	Patient's drug taken compliance in latest 1 month; Drug taken timing; Supervising doctor's visiting times; Health education given to patient.
	Lifestyle risk factors	Smoking; drinking; Intake of milk, tea and other diet habits, etc.
	Knowledge and faith	Patient's knowledge of TB curing, spreading, ADRs, etc; Patient's Faith in curing TB with current treatment.
Tuberculosis outcome record	Performance in the end of initial intensive phase of treatment	Whether patient's serum smear turns to be Mycobacterium negative. Whether a prolongation of initial intensive phase is needed.
	Time and reason for ending anti-TB therapy	Reasons: curing, exceeding 6-9 months, death form TB, death from other reasons, treatment failure, lost, outmigration, refusing treatment, misdiagnosis, or others.
ADACS follow up calendar	Discomforts	Everyday record of discomforting such as nausea, vertigo, headache, diarrhea, arthralgia, paresthesia, visual and auditory abnormal feelings.
	Anti-TB drug usage	Everyday record of usage of anti-TB drug.
Genotyping result	Candidate genes	N-acetyltransferase 2(NAT2); Cytochrome P450 2E1 (CYP2E1); Glutathione S-transferase (GST) enzyme(GSTM1, GSTT1), etc.

### Statistical methods

Incidence and prognosis of ADRs will be reported by descriptive statistics. Some conventional and special approaches such as multi-lever analysis and propensity score matching will be adopted for detecting environmental and genetic factors' associations with anti-tuberculosis induced ADRs and their interactions. The health economical data will be used for economical burden calculation and cost-effect analysis together with local socio-economic data.

### Ethical aspects

This study was approved by the ethics committee of Center for Tuberculosis Control and Prevention of China and Health Sciences Center of Peking University. Oral informed consent was obtained from every participant before their enrollment. Meanwhile written informed consent was signed by every study participant or surrogate. All participants would receive feedback reports and suggestions about their own health status after routinely laboratory examination, and participants with untreated conditions identified by the examination would also be referred to an appropriate healthcare provider.

To protect participants' privacy, no individual information, including genetic data would be informed. Personal identifiers, together with participants' name, Chinese ID card number, telephone number and home address would form a single database which only be used for contacting participants when further information needed. Every investigator who has access to field investigation data and genetic data is blinded to the PID database.

## Discussion

The ADACS adopted a standard longitudinal design, represented one of the largest and most diverse cohort of patients receiving WHO recommending anti-TB treatment. Thus, the major strength of this cohort is that our large sample size, diverse fields and intensive follow-up will enable us to determine rates of anti-TB induced ADRs accurately and generalize the result to similar populations under certain conditions.

As outcome variable, ADR's determination is very important. The previous studies of ADRs seldom did relationship assessment for suspicious drug and target ADRs, so sometimes misjudgment of ADRs' origin cause was inevitable. In this study, we cooperated with China SFDA, not only promoted it to establish a diagnostic criterion for ATLI recognition and grading, but also let every ADE reported receive strictly casualty assessment by SFDA to determine whether it was ADR or not. This will ensure the accuracy of ADR determining and avoid overestimating the incidences of ADRs.

We adopted county CDC as our main investigation locations. We employed CDC doctors as field investigator. We set timing of patient examination and follow-up fitting common anti-TB regimen time arrangement. We let drug taken supervisors be ADRs supervisors at the same time. All of these ensure our cohort high participation rate, retention rate and ADR promptly reporting rate.

The integration of multiple study methods is another feature of ADACS. For ADRs incidences estimation, we introduced systematic review, data mining and cohort study. In preparing phrase of the study, we wrote systematic reviews of anti-TB drug induced ADRs [12,33], analyzed data in ADR monitoring databases of China and WHO. We believe that could be a comparing source and supplement to our incidence estimation arisen from cohort data. For ADRs risk factors study, we implemented cohort study and case-control study. We measured environmental factors, genetic factors, and clinical factors and will analyze their independent effect and interactive effect. For ADRs impacts study, we searched ADRs' direct harms, their economical impacts, their impacts on TB treatment and TB curing, and their influences to TB epidemic control. These will let us view ADRs from a more comprehensive perspective.

There are two main weaknesses of this study. The first one is that we only set patient to do laboratory examinations at before and 1 month after TB treatment begins, while not arranged examinations at 1 week or 2 weeks. If a patient developed symptom within 1 month, he would be reported as having ADE and sent for further check promptly. But if a patient didn't have any signs or symptoms, and was only recognized and confirmed to be having an ADR because of test result abnormality at 1 month, the discovering time of ADR would be delayed. That would influence estimation of average ADR onset time and let it be later. We planned to calculate proportions of such cases and made certain adjustment to lower its influence as much as possible. The second one is the floating population. They will add uncertainty to follow up. Although we anticipated a rather high retention rate according to our following methods, floating population will still be a problem especially after they develop an ADR, they might be more likely to stop anti-TB therapy and be lost to follow up. In this case, ADR happening information would be recorded, but ADR treatment and prognosis data might be lost which would require special and continuous effort to get contact with the patient again.

## Abbreviations

ADACS: Anti-tuberculosis Drugs induced Adverse Reactions in China National Tuberculosis Prevention and Control Scheme Study; TB: tuberculosis; CNTS: China National Tuberculosis Prevention and Control Scheme; ADRs: adverse drug reactions; DNA: deoxyribonucleic acid; SFDA: State Food and Drug Administration; ATLI: anti-tuberculosis drug induced liver injury; WHO: World Health Organization; DOTS: directly observed treatment strategy; H: Isoniazid; R: Rifampicin; Z: Pyrazinamide; E: Ethambutol; S: Streptomycin; CDC: Center for Disease Control; GDP: gross domestic product; PPS: probability proportional to size; HBsAg: Hepatitis B Surface Antigen; ADEs: adverse drug events; CDR: Center for Drug Reassessment; ALT: Alanine transaminase; NAT2: N-acetyltransferase 2; CYP2E1: cytochrome P450 2E1; GST: glutathione S-transferase; PID: personal identifier.

## Competing interests

The authors declare that they have no competing interests.

## Authors' contributions

YYX drafted most of the manuscript. SYZ contributed to the design, acquisition of study data and revised the manuscript critically. DYH, FYL, XMW, YLY, YXC also contributed to the design and acquisition of study data. DHT, LZ, LZZ, WWG, DFC, LY, XTL contributed to the design of the research. HYW, PPH mainly contributed to the sample size calculation and randomized sampling process. YJH, FS joined in the design of investigation questionnaire. All authors have given final approval of the version to be published.

## Pre-publication history

The pre-publication history for this paper can be accessed here:

http://www.biomedcentral.com/1471-2458/10/267/prepub

## References

[B1] WHO Global tuberculosis control: epidemiology, strategy, financing 2009 P7 46 WHO document WHO/CDS/TB;

[B2] WHO An expanded DOTS framework for effective tuberculosis control.Stop TB Communicable Diseases 2002 1 20 WHO document WHO/CDS/TB; 12019913

[B3] Xianyi C Fengzeng Z Hongjin D Liya W Lixia W Xin D Chin DP The DOTS strategy in China: results and lessons after 10 years Bull World Health Organ 2002 80 6 430 436 12131998 PMC2567538

[B4] Awofeso N Anti-tuberculosis medication side-effects constitute major factor for poor adherence to tuberculosis treatment Bull World Health Organ 2008 86 3 B D 18368191 10.2471/BLT.07.043802 PMC2647396

[B5] Berg J Blumberg EJ Sipan CL Friedman LS Kelley NJ Vera AY Hofstetter CR Hovell MF Somatic complaints and isoniazid (INH) side effects in Latino adolescents with latent tuberculosis infection (LTBI) Patient Educ Couns 2004 52 1 31 39 10.1016/S0738-3991(02)00268-9 14729288

[B6] Combs DL O'Brien RJ Geiter LJ USPHS Tuberculosis Short-Course Chemotherapy Trial 21: effectiveness, toxicity, and acceptability. The report of final results Ann Intern Med 1990 112 6 397 406 2155569 10.7326/0003-4819-76-3-112-6-397

[B7] Nikolaeva OD [Side effects of chemotherapy in patients with pulmonary tuberculosis and concomitant diseases] Lik Sprava 2003 3-4 74 78 12889365

[B8] Kishore PV Palaian S Ojha P Shankar PR Pattern of adverse drug reactions experienced by tuberculosis patients in a tertiary care teaching hospital in Western Nepal Pak J Pharm Sci 2008 21 1 51 56 18166520

[B9] Snider DJ Long MW Cross FS Farer LS Six-months isoniazid-rifampin therapy for pulmonary tuberculosis. Report of a United States Public Health Service Cooperative Trial Am Rev Respir Dis 1984 129 4 573 579 6370060

[B10] Vieira DE Gomes M Adverse effects of tuberculosis treatment: experience at an outpatient clinic of a teaching hospital in the city of Sao Paulo, Brazil J Bras Pneumol 2008 34 12 1049 1055 10.1590/S1806-37132008001200010 19180340

[B11] Gulbay BE Gurkan OU Yildiz OA Onen ZP Erkekol FO Baccioglu A Acican T Side effects due to primary antituberculosis drugs during the initial phase of therapy in 1149 hospitalized patients for tuberculosis Respir Med 2006 100 10 1834 1842 10.1016/j.rmed.2006.01.014 16517138

[B12] Xia YY Zhan SY [Systematic review of anti-tuberculosis drug induced adverse reactions in China] Zhonghua Jie He He Hu Xi Za Zhi 2007 30 6 419 423 17673012

[B13] Kaplovitz N Drug-induced liver disorders: implication for drugdevelopment and regulation Drug Saf 2001 24 2 483 490 10.2165/00002018-200124070-00001 11444721

[B14] Bissell DM Gores GJ Laskin DL Hoofnagle JH Drug-induced liver injury:mechanisms and test systerms Hepatology 2001 33 9 1009 1013 10.1053/jhep.2001.23505 11283870

[B15] Wong WM Wu PC Yuen MF Cheng CC Yew WW Wong PC Tam CM Leung CC Lai CL Antituberculosis drug-related liver dysfunction in chronic hepatitis B infection Hepatology 2000 31 1 201 206 10.1002/hep.510310129 10613746

[B16] Schaberg T Rebhan K Lode H Risk factors for side-effects of isoniazid, rifampin and pyrazinamide in patients hospitalized for pulmonary tuberculosis Eur Respir J 1996 9 10 2026 2030 10.1183/09031936.96.09102026 8902462

[B17] Fernandez-Villar A Sopena B Fernandez-Villar J Vazquez-Gallardo R Ulloa F Leiro V Mosteiro M Pineiro L The influence of risk factors on the severity of anti-tuberculosis drug-induced hepatotoxicity Int J Tuberc Lung Dis 2004 8 12 1499 1505 15636498

[B18] Pande JN Singh SP Khilnani GC Khilnani S Tandon RK Risk factors for hepatotoxicity from antituberculosis drugs: a case-control study Thorax 1996 51 2 132 136 10.1136/thx.51.2.132 8711642 PMC473016

[B19] Shakya R Rao BS Shrestha B Incidence of hepatotoxicity due to antitubercular medicines and assessment of risk factors Ann Pharmacother 2004 38 6 1074 1079 10.1345/aph.1D525 15122004

[B20] Yee D Valiquette C Pelletier M Parisien I Rocher I Menzies D Incidence of serious side effects from first-line antituberculosis drugs among patients treated for active tuberculosis Am J Respir Crit Care Med 2003 167 11 1472 1477 10.1164/rccm.200206-626OC 12569078

[B21] Teleman MD Chee CB Earnest A Wang YT Hepatotoxicity of tuberculosis chemotherapy under general programme conditions in Singapore Int J Tuberc Lung Dis 2002 6 8 699 705 12150482

[B22] Ungo JR Jones D Ashkin D Hollender E S Bernstein D Albanese A P Pitchenik A E Antituberculosis drug-inducted hepatotoxicity: The role of hepatitis C virus and the human immunodeficiency virus AMJ Respir Crit Care Med 1998 15 7 1871 10.1164/ajrccm.157.6.9711039 9620920

[B23] Massacesi C Santini D Rocchi MB La Cesa A Marcucci F Vincenzi B Delprete S Tonini G Bonsignori M Raltitrexed-induced hepatotoxicity: multivariate analysis of predictive factors Anticancer Drugs 2003 14 7 533 541 10.1097/00001813-200308000-00005 12960737

[B24] Ozick LA Jacob L Comer GM Lee TP Ben-Zvi J Donelson SS Felton CP Hepatotoxicity from isoniazid and rifampin in inner-city AIDS patients Am J Gastroenterol 1995 90 11 1978 1980 7485004

[B25] Stern JO Robinson PA Love J Lanes S Imperiale MS Mayers DL A comprehensive hepatic safety analysis of nevirapine in different populations of HIV infected patients J Acquir Immune Defic Syndr 2003 34 Suppl 1 S21 S33 14562855 10.1097/00126334-200309011-00005

[B26] Nolan CM Goldberg SV Buskin SE Hepatotoxicity associated with isoniazid preventive therapy: a 7-year survey from a public health tuberculosis clinic JAMA 1999 281 11 1014 1018 10.1001/jama.281.11.1014 10086436

[B27] Ungo JR Jones D Ashkin D Hollender ES Bernstein D Albanese AP Pitchenik AE Antituberculosis drug-induced hepatotoxicity. The role of hepatitis C virus and the human immunodeficiency virus Am J Respir Crit Care Med 1998 157 6 Pt 1 1871 1876 9620920 10.1164/ajrccm.157.6.9711039

[B28] Dossing M Wilcke JT Askgaard DS Nybo B Liver injury during antituberculosis treatment: an 11-year study Tuber Lung Dis 1996 77 4 335 340 10.1016/S0962-8479(96)90098-2 8796249

[B29] Hwang SJ Wu JC Lee CN Yen FS Lu CL Lin TP Lee SD A prospective clinical study of isoniazid-rifampicin-pyrazinamide-induced liver injury in an area endemic for hepatitis B J Gastroenterol Hepatol 1997 12 1 87 91 10.1111/j.1440-1746.1997.tb00353.x 9076631

[B30] Lee BH Koh WJ Choi MS Suh GY Chung MP Kim H Kwon OJ Inactive hepatitis B surface antigen carrier state and hepatotoxicity during antituberculosis chemotherapy Chest 2005 127 4 1304 1311 10.1378/chest.127.4.1304 15821209

[B31] Wu JC Lee SD Yeh PF Chan CY Wang YJ Huang YS Tsai YT Lee PY Ting LP Lo KJ Isoniazid-rifampin-induced hepatitis in hepatitis B carriers Gastroenterology 1990 98 2 502 504 2295408 10.1016/0016-5085(90)90846-s

[B32] Ormerod LP Horsfield N Frequency and type of reactions to antituberculosis drugs: observations in routine treatment Tuber Lung Dis 1996 77 1 37 42 10.1016/S0962-8479(96)90073-8 8733412

[B33] Sun F Chen Y Xiang Y Zhan S Drug-metabolising enzyme polymorphisms and predisposition to anti-tuberculosis drug-induced liver injury: a meta-analysis Int J Tuberc Lung Dis 2008 12 9 994 1002 18713495

[B34] Liu Q Garner P Wang Y Huang B Smith H Drugs and herbs given to prevent hepatotoxicity of tuberculosis therapy: systematic review of ingredients and evaluation studies Bmc Public Health 2008 8 365 373 10.1186/1471-2458-8-365 18939987 PMC2576232

[B35] WHO Tuberculsis fact sheets 2007 http://www.who.int/mediacentre/factsheets/fs104/en/

[B36] Provincial gross domestic product summary in 2007 Chinese National Bureau of Statistics. National Accounts: 3-13. 2008 http://www.stats.gov.cn/tjsj/ndsj/2007/indexeh.htm

[B37] Tack LC Thomas M Reich K Automated forensic DNA purification optimized for FTA card punches and identifiler STR-based PCR analysis Clin Lab Med 2007 27 1 183 191 10.1016/j.cll.2006.12.009 17416311

[B38] World Health Organization WHO UMC The use of the WHO-UMC system for standardized case causality assessment 2007 http://www.who-umc.org/graphics/4409.pdf

